# Influence of learning styles on student performance in self-instructional courses

**DOI:** 10.1371/journal.pone.0289036

**Published:** 2023-07-27

**Authors:** Alana Oliveira, Vitoria Spinola, Deise Garrido, Mario Meireles Teixeira, Carlos Salles, Ana Estela Haddad

**Affiliations:** 1 Computer Engineering, Federal University of Maranhao, Sao Luis, MA, Brazil; 2 USP School of Dentistry, University of Sao Paulo, Sao Paulo, SP, Brazil; 3 Department of Informatics, Federal University of Maranhao, Sao Luis, MA, Brazil; Federal University of Minas Gerais, Nursing School, BRAZIL

## Abstract

This study aimed to analyze the learning style of dentistry students in self-instructional courses to assist in pedagogical planning and to choose the most appropriate educational resources for the students’ learning profile. A sample of 122 students who responded to the Learning Styles Questionnaire was analyzed. For statistical purposes, correlation analysis, chi-square test, odds ratio, and Student’s t-test were performed. In the analyzed sample, there was a higher prevalence of students in the theoretical and reflector styles, and a lower prevalence of students in the activist and pragmatic styles. An analysis of educational resources demonstrated the predominance of theoretical and reflective content. The data show a statistically significant reduction of about 74% in the chances of passing for the activist-pragmatists group compared to other students (*χ*^2^(1, N = 122) = 5.795, p < 0.05, odds ratio = 0.26). On the other hand, reflector students who exhibited a lower preference for the activist style had a higher chance of course completion, with a 3.33-fold increase in the likelihood of passing the course (*χ*^2^(1, N = 122) = 5.637, p < 0.05, odds ratio = 3.33). These findings highlight the importance of considering students’ learning styles in educational planning and resource selection to optimize student performance. Further research is warranted to explore the implications of these findings and to investigate additional factors that may influence student success in self-instructional courses.

## Introduction

Health education is a multifaceted field in which different conceptions converge, reflecting different understandings of the world and requiring a vision of different sciences. From this perspective, it is important to rethink the concepts on which higher education is based, whose central point is no longer teaching but the teaching-learning process [1, 2].

Autonomous learning gives more importance to the mastery of teaching tools than mere accumulation of content, starting with educational resources capable of generating significant knowledge and facilitating the insertion of students in the learning process [3]. Learning analytics provide tools for understanding and optimizing learning and the environment in which it happens [4, 5].

Among the characteristics of the teaching process, learning styles stand out in this article, where mechanisms capable of classifying students according to the most favorable way for their learning are sought; that is, identifying the most appropriate way for students to learn. The challenges range from understanding how each student learns best to the volume and nature of the content to be offered, its frequency, and the display of what is relevant–that is, offering personalized teaching at massive levels. Therefore, collecting data on student profiles, particularly their learning styles, is an important step in any data-driven decision-making process to assist in planning and executing massive open online courses (MOOCs).

The relationship between learning styles, academic performance, and adequacy of instructional materials has received significant attention in educational research. Previous studies suggest that students have different preferences and ways of absorbing and processing information, which can impact their academic performance. By understanding and considering students’ learning styles, it is possible to adapt instructional materials and teaching strategies to better meet individual needs. This creates a more personalized and engaging learning environment, providing students with greater opportunities for academic success. Therefore, investigating the relationship between learning styles, academic performance, and adequacy of instructional materials has significant implications for the development of more effective educational approaches and for enhancing the quality of students’ learning experience.

It is clear that teachers are not advised to diagnose every student. On the other hand, attention to learning styles brings about a variety of teaching practices. Information regarding students, their backgrounds, experiences, and learning styles can be effectively used in the classroom [6], whether face-to-face or distant. However, the choice of learning style is not unanimous, as pointed out by [7, 8].

In a study, Lowery [9] points out resources and specificities to be used in teaching and assessment in order to contemplate all learning styles in the classroom. Different media types are perceived differently by students according to their learning styles and Lowery state that the right selection of educational media can improve students’ learning achievement.

In a related work, we propose adaptations in the interface of a virtual environment according to the students’ learning style, thus seeking to provide a differentiated and potentially more motivating user experience [10] and to improve students’ performance as indicated by [11].

In this work, it is assumed that a more accurate mapping between students’ learning preferences and the educational content provided has the potential to increase their performance in carrying out courses. It should be noted that the purpose of learning styles is not to match the instruction provided to each student’s learning style preferences, but rather to teach in a way that balances students’ preferences for different learning styles [12]. This approach can be applied to different educational contexts.

Particularly, self-instructional courses have been used since 2010 to train professionals in the Unified Health System (SUS), the Brazilian Public Health System, through the Open University of SUS (UNASUS). Since 2016, UNASUS/UFMA, in partnership with NuTes FOUSP, the Teledentistry and Telehealth Center of the School of Dentistry at the University of São Paulo (FOUSP), have been developing MOOCs for the continuing education of SUS dentists.

Our research aimed to analyze the learning style of students of self-instructional courses in the area of dentistry to assist in the pedagogical planning of these courses and to select the most appropriate educational resources directed toward the students’ learning profile. To this end, we conducted a narrative review of the literature, focusing on key articles that discuss the application of learning styles and their impact on performance in online courses. This review served to provide a theoretical foundation for our study and to identify relevant trends and gaps in the current body of knowledge.

This study aimed to (i) Identify the learning profile of the course’s target audience; (ii) Identify the profile of the course’s educational resources; (iii) Perform a compatibility analysis of the educational resources present in the course with the students’ profile.

The insights gained from this work have the potential to contribute to the continuous development of the pedagogical team at UNASUS/UFMA. This research offers valuable perspectives, encouraging a thoughtful evaluation of existing pedagogical approaches and suggesting avenues for further growth.

## Theoretical background

### Learning styles

Each human being has their own way of learning, those who learn by doing and those who observe, those who are multitasking, and those who need to focus on one task at a time. Several studies intend to classify students according to the way most favorable to their learning, that is, according to their learning style, seeking to identify the best way for a student to assimilate the knowledge that is transmitted.

#### Kolb’s learning styles and experiential learning cycle

For Kolb [13, 14], learning is the process by which knowledge is created through the transformation of experience. And knowledge is not something that can simply be transmitted or acquired, it is the result of a process and can be created and recreated continuously. Kolb also believes that people can be classified according to their way of learning into learning styles (or preferences) as diverging, converging, assimilating, and accommodating. This classification can be used to provide teachers with information so that they consider the best way their students can learn and thus be able to achieve better success in their teaching.

#### Honey and Mumford learning styles

Honey and Mumford have identified, based on Kolb’s work, four learning styles or preferences: Activist, Theorist, Pragmatist, and Reflector. The authors recommend that, to maximize personal learning, each student should know their own learning style and then look for opportunities to learn using that style.

The four learning styles characterized by Honey and Mumford are activist, reflective, theorist and pragmatic [15]. Activists do not show prejudice when entering new experiences nor do they show the ability to work with people. Reflectors show a preference for looking at experiences from different angles and need time to reflect on their circumstances before establishing a course of action. According to theorists, learning depends on their understanding of concepts, models, and theories, presenting their ability to synthesize and analyze information. Finally, pragmatists must understand the practical benefits of applying theory in the real world, and feel comfortable following a predefined course of action.

## Related work

The relationship between students’ learning styles and academic performance has been an ongoing topic of interest across various fields of study. Numerous studies have explored this relationship across a variety of contexts and through diverse educational modalities, from traditional in-person education to emerging modalities enabled by advancements in technology. These include distance education, online learning, blended learning, and flipped classrooms, among others.

Regardless of the context or modality, a common theme emerges in the literature: the importance of tailoring instruction to the student’s learning style. A body of research suggests that by aligning the teaching strategy with the student’s preferred learning style, one can enhance student engagement, understanding of material, and ultimately, academic performance [16, 17].

However, it’s important to note that the relationship between learning styles and academic performance is not simple or direct. Some studies suggest that different learning styles may be more or less effective in different contexts or subjects. An illustrative example of this complex relationship between learning styles and academic performance is found in the study conducted by [16]. They applied the Kolb’s learning style model, to investigate this relationship within the context of an accounting course for non-accounting students. The findings revealed that students favoring the Pragmatist and Theorist learning styles outperformed their peers in the accounting course, while those with a preference for the Activist learning style lagged behind.

In [18], Shamsuddin and Kaur conducted a study exploring the impact of blended learning on student outcomes in relation to their learning styles. They employed Kolb’s Learning Style Inventory to determine the learning styles of 119 students pursuing a degree in Information Technology. The study primarily found a majority of students falling into the Convergent learning style, followed by Divergent, Accommodator, and Assimilator. However, the research did not uncover a meaningful correlation between students’ learning styles and their perceptions of blended learning.

EL-BISHOUTY et al. investigated the application of the Felder and Silverman learning style model in designing online courses [17]. They developed an interactive course analyzer tool that helps teachers assess the level of support provided by their courses for different learning styles. The findings indicated that designing courses with specific learning styles in mind can enhance learning outcomes for students with those particular styles.

## Materials and methods

### Study design and instruments

This was a descriptive, cross-sectional, quantitative study. In this article, a methodology centered on learning styles is presented according to the Honey-Mumford taxonomy [19, 20].

It is important to mention that reliance on participants’ self-reports may introduce inherent subjectivity and potential biases into the data collection process. We acknowledge the potential for discrepancies between self-perceptions and actual learning characteristics, as well as the influence of motivational factors on students’ perceptions and study decisions. This limitation is not unique to our study though but is a common challenge in research that relies on questionnaires or self-assessment tools. However, Flemimg and Baume [21] believe that the use of questionnaires to define learning styles can be useful, but its real value lies in the self-knowledge it can generate to each person when analyzing the score obtained. This classification can serve as input for teachers so that they can choose the most appropriate activities for each modality.

The analysis was based on secondary data collected directly from LMS Moodle and primary data obtained from the Learning Styles Questionnaire (LSQ) [22, 23]. In summary, the LSQ consists of 80 yes/no answer questions, allowing respondents to indicate their agreement with each statement that aligns with their learning preferences. The questionnaire covers various aspects related to learning approaches and behaviors associated with the four learning styles: Activist, Theorist, Pragmatist, and Reflector.

Upon completion of the LSQ, a score is obtained for each learning style. This score reflects the student’s intensity or preference level for that particular style. The scoring ranges from very low preference to very strong preference. By analyzing the responses, we were able to determine the dominant learning style preferences of the participants in our study.

It is important to note that the LSQ has been widely used and recognized as a valid and reliable instrument for assessing learning style preferences. The questionnaire items are based on theoretical constructs and empirical evidence related to the different learning styles proposed by Honey and Mumford. This information serves as input for checking the compatibility analysis of the educational resources present in the course, in relation to the students’ profile.

Finally, the chi-square test is carried out in order to investigate whether there is an association between learning styles and passing this course. By performing the student’s t test, it is verified whether there is a statistically significant difference between the performance (final grade) of students with a profile compatible with the course and those with an incompatible profile.

### Sample and data collection

The data collected were from students of the course “Dental Care for Patients with CNCDs in Primary Care: Diabetes, Hypertension and Chronic Kidney Disease”, which addresses topics related to healthcare networks for people with chronic diseases, especially chronic kidney disease (CKD), systemic arterial hypertension (SAH), and diabetes mellitus (DM).

The course was offered in three editions, with the first and second editions having 7,618 and 5,471 students, respectively. The third edition had a total of 19,958 enrolled students. For the purposes of this study, the participants invited to respond to the questionnaire were those who had already completed the course, totaling 8,763 students, including both those who passed and those who failed. Invitations were sent via email along with informational materials explaining each learning style, a list of activities more favorable or unfavorable for each style, and recommendations for suitable materials based on learning style. The questionnaire was made available for a period of one month, during which 225 students had access to the form. However, only 122 students completed the questionnaire in its entirety.

### Variables

Students were categorized as theorists, reflectors, pragmatists, and activists, after having completed a questionnaire made available as a web tool, applied to groups of students of a self-instructional course.

According to the number of responses in agreement, the preference for each learning style was determined on five levels: very low, low, moderate, high, or very high.

### Statistical analysis

Data were analyzed using version 28 of the Statistical Package for the Social Sciences (SPSS). Correlation analysis, chi-square, odds ratio and student’s t test were performed and the significance level was set at 5% (p < 0.05).

A statistical correlation analysis was conducted to identify profiles that overlapped. A chi-square test was conducted to investigate whether there was an association between learning styles and passing the course. The Student’s t-test was used to verify whether there was a statistically significant difference between the performance (final grade) of students with a profile compatible with the course and those with an incompatible profile. While the data did not follow a normal distribution, we chose to use the t-test with bootstrap resampling (1000 samples; 95% BCa confidence interval) as a robust alternative to test for differences between groups. Bootstrap resampling is a well-established method for generating reliable estimates and confidence intervals, even when the underlying data distribution is unknown or non-normal [24–26]. As such, we believe that the results obtained through this approach are valid and can provide useful insights into the relationship between the variables under study.

## Ethical statement

This project was approved (approval no. 3.809.169) by the Research Ethics Committee of the Universidade Federal do Maranhao (UFMA).

All participants of this research have expressed their consent electronically by agreeing to a consent form available on the Moodle LMS platform. All participants’ data were fully anonymized prior to the analysis.

## Results

### Matching types of resources to learning styles

The course addresses topics related to the Health Care Networks for People with Chronic Diseases, in particular Chronic Kidney Disease (CKD), Systemic Arterial Hypertension (SAH) and Diabetes Mellitus (DM). Having as an educational objective the understanding of the epidemiology of these chronic diseases as well as the diagnosis, treatment and dental management, in order to contribute to a better service, respecting the specificities and needs of this line of care.

This course is comprised of 14 resources, including welcome video, course expectation collection form, pre-test and post-test status questionnaires, pre- and post-test benchmarking and evaluation. Those resources are common to all courses and not specific educational content unique to the dentistry course under investigation.

The number of resources by learning style for each topic in the analyzed course is summarized (Table 1). Table summarization was based on the interface adaptation model presented in [10], which lists the most appropriate types of educational resources for each learning style. Activist students benefit from resources, such as competition, challenges, movies, forums, infants, games, and mind maps. Reflectors preferred handouts, digital books, articles, documentaries, movies, exercise lists, podcasts, riddles, video classes, and webcasts. Theorists prefer handouts, digital books, articles, documentaries, video classes, video demos, and webcasts. Finally, pragmatists were most comfortable with documentaries, movies, infographics, games, workbooks, and tutorials. It is clearly observed that the educational resources produced for the analyzed course are directed only to the theoretical and reflector profiles.

**Table 1:** Number of resources by learning style in the course.

#	Topics	Activist	Reflector	Theorist	Pragmatist
1	Chronic non-communicable diseases and oral health care in Primary Health Care	0	0	0	0
2	Dental care of patients with CKD	0	1	1	0
3	Dental care for patients with hypertension	0	1	1	0
4	Dental care for patients with diabetes	0	1	1	0
5	Dental care for people with sickle cell disease	0	0	0	0
	Total	0	3	3	0

### Sample characterization

**Fig 1:**
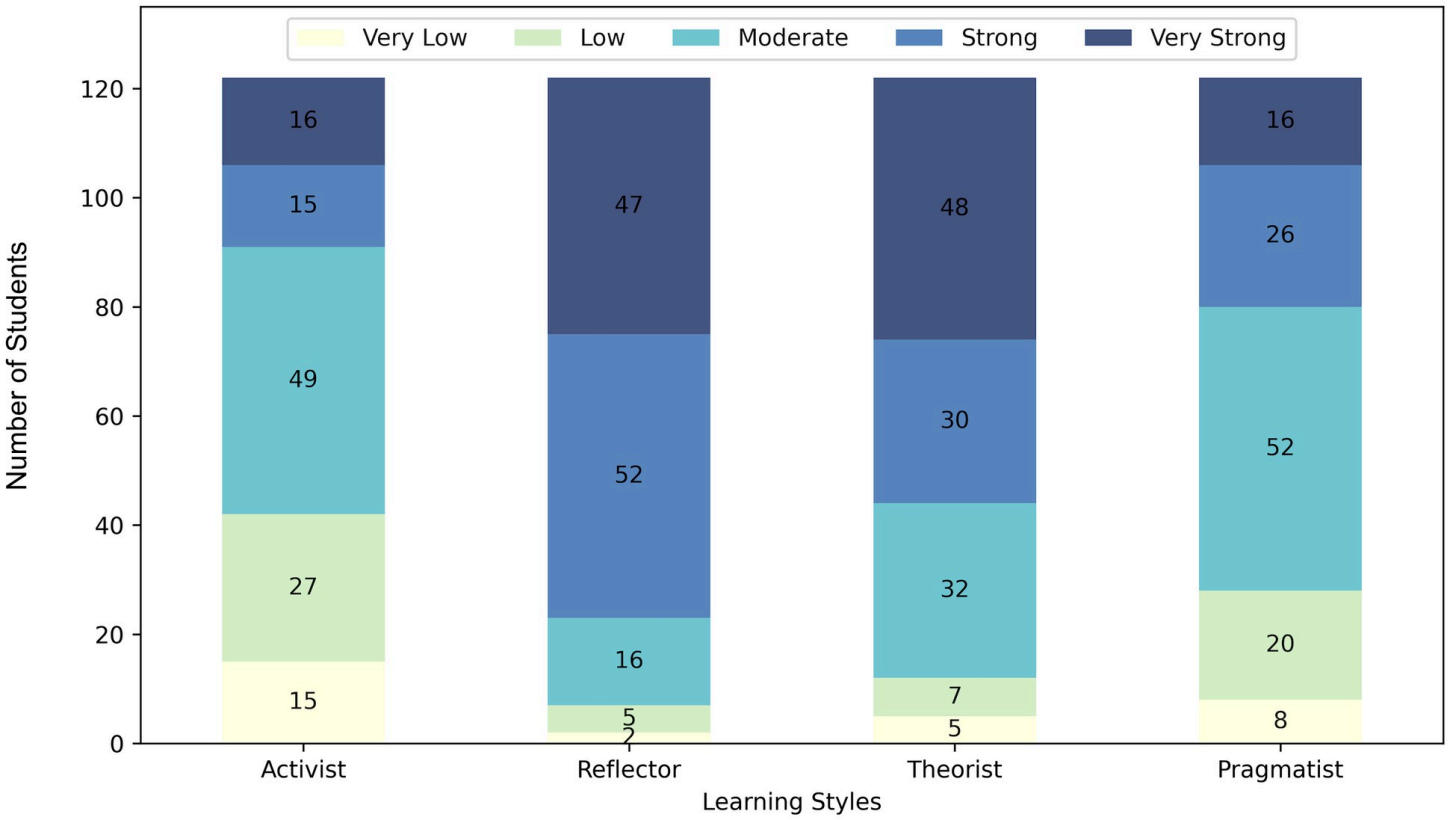
Proportion of students by preference level in each learning style.

The graph quantifies the students according to their level of preference for each learning style (Fig 1). Reflector style was predominant, with approximately 81% (99/122) showing a high or very high preference. The smallest trend was for the activist style, with only 25.4% (31/122) of participants at high and very high preference levels.

### Statistical correlation between learning styles

**Fig 2:**
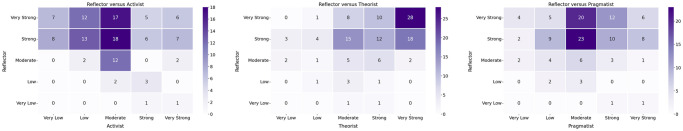
Cross-frequency heatmap of reflectors students.

The reflector learning style is crossed with other styles (Fig 2). In it, it can be seen that the first heat map (Fig 2a) highlights a moderate to very low activist preference combined with a strong to very strong preference for reflectors in 61.47% (75/122) of the participants. Additionally, when cross-referenced with theorist learning style (Fig 2b), the heat map shows a concentration of strong and very strong reflector preference combined with an increasing moderate to very strong theorist preference in 74.59% (91/122) of participants. This indicated a combined preference for these two styles among the participants. A similar behavior occurred when the cross between reflexive and pragmatist (Fig 2c) was verified, with a strong and very strong reflexive preference combined with a moderate to very strong pragmatist preference in 64.75% (79/122) of the participants.

**Fig 3:**
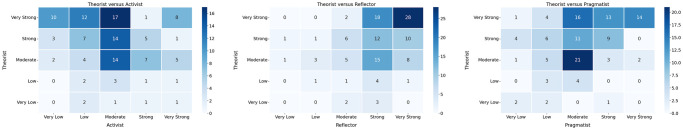
Cross-frequency heatmap of theorist students.

A moderate to very low activist preference is noticed, combined with a moderate to very high preference of theorists in 68.03% (83/122) of the participants (Fig 3a).

**Fig 4:**
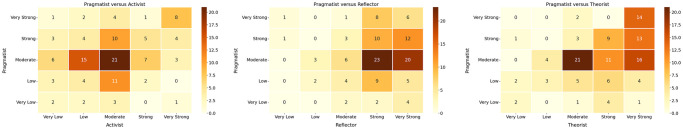
Cross-frequency heatmap of pragmatists students.

Finally, a centrally distributed preference between activists and pragmatists stood out in 64.75% (79/122) of the participants (Fig 4).

The obtained correlation coefficients were analyzed (Table 2). The variables “activist”, “pragmatist”, and “theorist” are significantly correlated (p < 0.01 and p < 0.05). The variable “reflector” was statistically correlated only with the “theorist” learning style (p < 0.01). Statistical analysis of the learning styles showed a positive correlation between reflectors and theorists, theorists, and pragmatists, and between activist and pragmatist styles. A significant negative correlation between the activist and theorist styles was also observed for the studied sample.

**Table 2:** Correlation coefficients between learning styles.

Variables	Activist Level	Reflector Level	Theorist Level	Pragmatist Level
Activist Level	-			
Reflector Level	-0,161 (n.s)	-		
Theorist Level	-0,215*	0,392**	-	
Pragmatist Level	0,283**	0,095 (n.s)	0,409**	-

Note:

*p<.05;

**p<.01;

n.s. = non-significant correlation

### Association between learning styles and approval

Chi-square tests of independence (2x2) were performed (Table 3) to investigate whether there was an association between learning style (reflectors, theorists, activists, and pragmatists) and passing the course. Thus, significant associations were found between the activist and pragmatist styles and passing the course.

**Table 3:** Result of the chi-square learning styles vs. passing test.

Learning Styles		pass/fail test		*χ*^2^ (dof)	Odds Ratio
		Yes	No		
Reflector	No	22	1	2.440 (1)	0.219
	Yes	82	17		
Theorist	No	37	7	0.073 (1)	1.152
	Yes	67	11		
Pragmatist	No	72	8	4.176 (1) **	0.356
	Yes	32	10		
Activist	No	82	9	6.737 (1) **	0.268
	Yes	22	9		
Activist-Pragmatist	No	92	12	5.795 (1) **	0.261
	Yes	12	6		
Reflector-low-Activist	No	24	9	5.637 (1) **	3.333
	Yes	80	9		

Note:

**p<0.05;

*χ*^2^ = chi-square; dof = degrees of freedom;

For the activist style, chi-square (*χ*^2^ (1) = 6.737, p = 0.009; Φ = -0.235) and odds ratio analyses showed that activist students experienced a reduction of 73.17% in the chance of passing the analyzed course compared to the non-activist ones. This is the same as saying that students who are not activists are 3.73 times more likely to pass the course when compared to activist students.

As for pragmatist style, the chi-square (*χ*^2^(1) = 4.176, p = 0.041; Φ =-0.185) and odds ratio analysis showed that pragmatist students had a 64.44% reduction in the chance of passing the analyzed course when compared to non-pragmatist students. This is the same as saying that students who are not pragmatist are 2.81 times more likely to pass the course when compared to pragmatist students.

Another chi-square test of independence (2x2) was conducted, considering the combination of activist and pragmatist styles, which are considered incompatible with the course, to investigate whether there was an association between the combination of these two styles and student approval. A significant association was found between these combined styles (activist-pragmatist) and approval (*χ*^2^(1) = 5.795, p < 0.05; Φ = 0.218). Odd-ratio analysis showed that activist-pragmatist students had a 73.91% reduction in the chance of passing the analyzed course compared to non-activist-pragmatist students.

On the other hand, reflector students with a low preference for the activist style are 3.33 times more likely to pass the course when compared to those who are not “low activist reflectors” (*χ*^2^(1)= 5.637, p < 0.05; Φ = -0.215).

This suggests that being “reflective low-active” is associated with a higher likelihood of course approval. However, it is important to note that this is an association and does not necessarily imply causation. Other factors not included in this study may also influence these results.

### Impact of learning style on course performance (final grade)

Additionally, a Student’s t-test was performed for independent samples (Table 4) to investigate the extent to which course performance (final grade) differed between students with a learning style consistent with the course and those who did not. The results showed that active students had statistically lower performance (M = 72.26; SD = 18.923) than non-active students (M = 80.00; SD = 14.832) (t(120) = -2.33, p < 0.05). The effect size of the difference was medium (Cohen’s d = 0.49).

**Table 4:** Activist performance difference test results (yes or no).

Groups	Grade	T test Statistics						
	Mean	SD	t	dof	p-value	Mean Diff	95% Confidence Interval of the Difference	
							Lower	Upper
Activist Yes	72.26	18.92	-2.33	120	0.021	-7.74	-14.31	-1.17
Activist No	80	14.83						

Note: SD = standard deviation; dof = degrees of freedom;

## Discussion

Health education encompasses a broad range of factors that extend beyond the mere acquisition of knowledge passed down from previous generations. They must also be able to transpose such knowledge for use in situations of daily professional practice. In the context of self-instructional distance courses (MOOCs), this task seems even more challenging because, most of the time, these courses provide theoretical material with few activities that approach the problems of practice.

Pedagogically, some studies categorize individuals according to learning styles, grouping common characteristics that allow a better understanding of how each student learns, how they receive and interact with different content. This approach becomes even more crucial when designing instructional strategies for massive courses. In line with this, our analysis examined the distribution of course resources based on learning styles. Furthermore, the findings revealed that the materials provided in the course predominantly aligned with the reflector and theorist profiles (Table 1), while the activist and pragmatic profiles were not adequately addressed. This discrepancy highlights the need for a more comprehensive and inclusive approach to cater to the diverse learning preferences of students in massive courses.

When examining the learning profile of the course’s target audience,it is noteworthy that a significant proportion of the students (approximately 81% and 64% of the participants, respectively) exhibit a preference for the reflector and theorist learning styles. Moreover, the pragmatist style also demonstrates a notable presence among the students to some extent (Fig 1). These findings indicate that the majority of the researched students display moderate to very high levels of preference for these particular learning profiles. There is also shows a moderate to very low activist preference combined with a moderate to very strong theorist preference for 90% of the participants (Fig 3). Therefore, our findings have demonstrated a certain degree of orthogonality between the students’ preferences regarding the theorist and activist profiles, which was evidenced visually and by statistical correlation analysis.

In general, education in Brazil aims to provide a solid foundation of theoretical knowledge, but there is also a growing recognition of the importance of more practical and contextualized approaches to promote meaningful learning and the development of relevant skills for the modern world [27], with little emphasis on practice, which leads individuals to develop such learning preferences over others. This emphasis on theory may have shaped the learning preferences of individuals, potentially contributing to the prevalence of reflector and theorist styles in our sample. However, this does not mean that they could not take advantage of didactic material that proposes more practical activities; they just were not trained to do so.

We investigated whether students’ learning styles influence their chances of passing the course, building upon the previous findings regarding the prevalence of learning styles in our sample. The results of the chi-square analysis and odds ratio analysis indicate a strong correlation between having an activist, pragmatist, or combined profile and a reduced likelihood of passing the analyzed course, with approximately a 73% decrease in the chances of passing. On the other hand, it was shown that students with a reflector and not a very active profile were more than three times more likely to pass than those with divergent profiles. We believe that this occurs precisely because of the non-conformity of the predominant learning style in these students with that identified in the educational resources. This demonstrates the importance of having varied resources to meet different student profiles and to develop their skills in different shades.

In addition, through Student’s t-test, it was found that the final grade of activist students in the course differed between students with a learning style compatible with the educational resources used and those who were not. Students with an activist profile presented a statistically lower performance than those with a non-activist profile.

In Tahir et al.’s study, the Pragmatist and Theorist styles were linked to superior performance in an accounting course. In our study, students with theoretical and reflective learning styles performed better. This discrepancy might be due to differences in the nature of the subjects studied—accounting and dentistry—suggesting that the impact of learning styles can vary depending on the discipline.

Contrasting Shamsuddin and Kaur’s (2020) findings, our study links student learning styles significantly to academic performance in self-instructional courses. While the previous study did not find a substantial correlation between learning styles and perceptions of blended learning, our findings reveal a significant association between learning styles and course outcomes.

The results in EL-BISHOUTY et al. (2019) suggest that considering students’ learning styles in course design can enhance learning outcomes for those specific styles. This finding aligns with our study, which indicates a significant association between learning styles and students’ performance in dentistry courses.

## Conclusion

In this study, we collected data on the learning styles of students in a self-instructional course in dentistry offered by UNASUS/UFMA in partnership with NuTes FOUSP. By categorizing the educational resources of the course according to students’ learning styles, we were able to identify profiles of students with the highest probability of success in the course. Our statistical analysis revealed that students with educational resources that were compatible with their learning styles had significantly higher pass rates than those with incompatible profiles.

These findings are consistent with previous research that has shown a strong relationship between learning styles and academic success. In particular, studies have shown that students who receive instruction that is tailored to their learning style tend to perform better than those who do not. Our study contributes to this body of literature by providing evidence that this relationship holds true in the context of a self-instructional course in dentistry.

However, it is important to note that our study has some limitations. For example, we relied on self-reported measures of learning styles, which may not accurately capture the complexity of individual learning preferences. Additionally, our sample was limited to a single course at a single institution, which may limit the generalizability of our findings.

Despite these limitations, our study has important implications for the design and delivery of educational content in the context of self-instructional courses. By considering the learning styles of students and tailoring educational resources to their preferences, it may be possible to improve student engagement, satisfaction, and ultimately, academic success.

This perspective is supported by our results, which indicate that by understanding the student’s learning style and adjusting the educational content to that style, whether through automatic adaptive means or through recommendations to students and teachers, academic performance can be improved overall.

Future research could build on these findings by exploring the effectiveness of different approaches to tailoring educational content to individual learning styles, as well as the factors that may influence the relationship between learning styles and academic success in different contexts.

This study presents opportunities for improving the development of educational content for new self-instructional courses that are better tailored to the target audience. In future work, we aim to expand this study to encompass other dentistry courses and areas in order to validate the identified trends.

Further investigations can explore the relationship between learning styles and additional measures of student success, such as retention rates or academic performance in subsequent courses. This would allow for a more refined design of educational content and support services to better cater to the diverse needs of learners.

Conducting longitudinal studies can provide insights into how learning styles evolve over time and their correlation with long-term academic and professional outcomes. This approach can offer valuable information about the factors that contribute to student success and inform the development of personalized educational programs.

Utilizing advanced statistical techniques, such as machine learning algorithms, can reveal patterns and relationships in extensive datasets of student performance and learning style assessments. This approach can uncover new insights and predictive models to improve the design and delivery of educational content.

Qualitative research methods, such as interviews or focus groups, can provide in-depth insights into how students with different learning styles experience and engage with educational content. This approach can identify specific challenges and opportunities for enhancing the design and delivery of educational materials and support services.

Lastly, it is crucial to develop and test new interventions or technologies that are specifically tailored to the needs of different learning styles. Examples include interactive tutorials or simulations, personalized feedback and coaching, and adaptive learning platforms that dynamically adjust content based on individual learners’ needs and preferences.

## Supporting information

S1 Data (ZIP)

S1 File (PDF)
