# Supplementary material for: Influence of learning styles on student performance in self-instructional courses
Source: PLoS One. 2023 Jul 27;18(7):e0289036. doi: 10.1371/journal.pone.0289036 (PMC10374043; doi:10.1371/journal.pone.0289036)
Supplement: S1 File — (PDF) [file pone.0289036.s002.pdf]

Supporting information

The supporting the conclusions of this article is included within the article and its additional files (available at the following link: https://github.com/alana-ufma/lrng-styles-dataset).
